# Hidradenitis Suppurativa: A Review of the Biologic and Small Molecule Immunomodulatory Treatments

**DOI:** 10.1177/12034754241300292

**Published:** 2024-11-27

**Authors:** Nicholas Chiang, Raed Alhusayen

**Affiliations:** 1 Temerty Faculty of Medicine, University of Toronto, Toronto, ON, Canada; 2 Department of Dermatology, Sunnybrook Health Sciences Centre, Toronto, ON, Canada

**Keywords:** hidradenitis suppurativa, biologic, immunomodulator

## Abstract

Hidradenitis suppurativa (HS) is a chronic inflammatory skin disease that presents as painful, deep-seated nodules, sinus tracts, and abscesses in about 1% of the population. Although the pathogenesis of HS is not perfectly understood, it is generally recognized to be caused by a combination of genetic, endocrine, environmental, and microbiological factors. The treatment principles of HS focus on decreasing the microbial load with antibiotics and/or modulating the host immune response to reduce inflammation. The treatment of adults with moderate-to-severe HS has significantly changed recently with the development of new biological medications and immunomodulators. While previously the mainstay of treatment of moderate-to-severe HS was adalimumab, a biologic tumour necrosis factor α inhibitor, the evidence for the use of other treatment classes such as interleukin (IL)-17 inhibitors, IL-1 inhibitors, and Janus kinase inhibitors has been growing. The goal of this review article is to review the available evidence that supports the efficacy and safety of biologics and small molecule immunomodulator treatments to treat adults with moderate-to-severe HS.

## Introduction

Hidradenitis suppurativa (HS), also known as acne inversa or Verneuil’s disease, is a chronic, recurrent, inflammatory skin disease that affects about 1% of the population.^1,2^ HS typically presents with painful, deep-seated nodules, sinus tracts, and abscesses in the intertriginous areas such as the axillae, inframammary, groin, and perineal regions.^1,3^ It typically manifests in the 3rd decade of life, however, its onset has also been described in children and post-menopausal women.^1,2,4^ Although the pathogenesis of HS is not perfectly understood, it is generally recognized to be caused by a combination of genetic, endocrine, environmental, and microbiological factors.^4,5^

The treatment of HS is challenging and has been described in several guidelines and articles.^6,7^ In short, treatment strategies focus on decreasing the microbial load with antibiotics and/or modulating the host immune response to decrease inflammation. These guidelines excellently summarize the treatment options for mild-to-moderate HS; however, they do not include new evidence for the treatment of moderate-to-severe HS.^6^ In the last decade, the body of evidence for new treatments for moderate-to-severe HS has significantly grown. Understanding what treatment options are available and the evidence to support these medications is necessary to ensure that patients with moderate-to-severe HS have optimal medical management.

## Materials and Methods

The goal of this scoping review is to evaluate the available evidence that supports the efficacy and safety of biologics and small molecule immunomodulator treatments to treat adults with moderate-to-severe HS. Although surgical and procedural interventions are an important strategy in the management of HS treatment, they fall outside the scope of this review and are reviewed elsewhere.^8^

**Figure 1.**
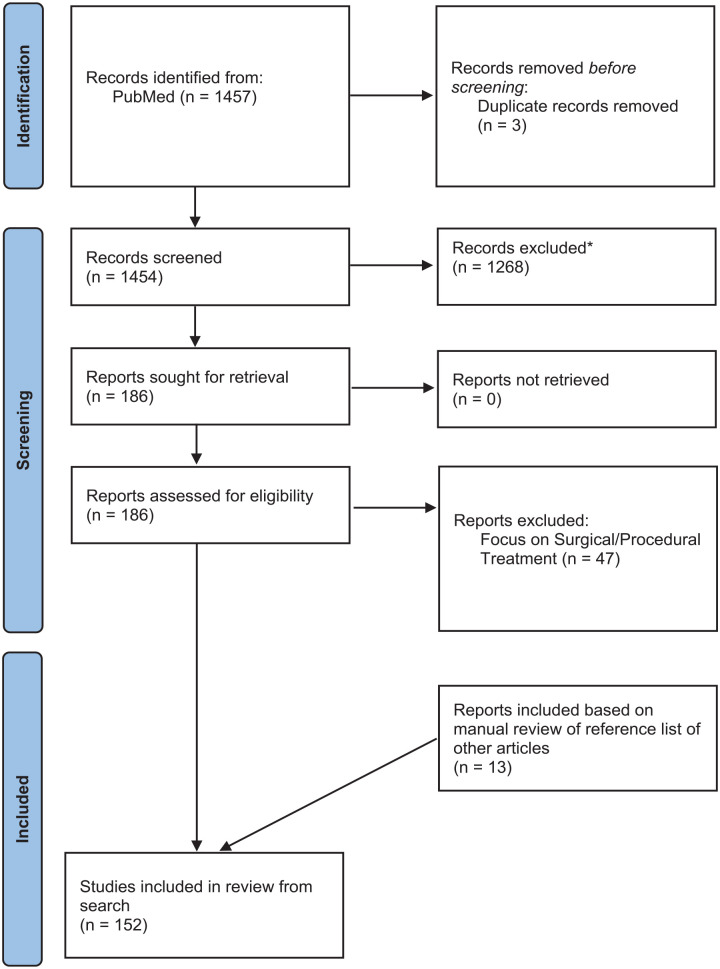
PRISMA flow diagram. *Reasons for excluding records: Records not about HS, records not about the treatment of HS, records about special populations of HS (paediatrics, pregnancy, etc.), records about the surgical and procedural management of HS. HS, Hidradenitis suppurativa.

We searched the MEDLINE medical database from inception until February 1, 2024. Variations of the following keywords were used for the search: “hidradenitis suppurativa,” “biologic,” “immunomodulatory,” and “treatment.” Titles, abstracts, and full text articles were screened by 1 author (N.C.). Inclusion criteria were the following: studies reporting on the treatment of moderate-to-severe HS with either biologic or small molecule immunomodulator treatments modalities. Studies were excluded if the focus of the article was not about the treatment of moderate-to-severe HS, the article focused on special populations of HS (paediatrics, pregnancy, etc.), and articles about the surgical and procedural management of HS. In addition, we manually searched the reference list of relevant included articles to identify gaps in our search. One hundred fifty-two articles were deemed eligible for inclusion in this scoping review (see Figure 1).

## Treatment Outcomes in HS

### Treatment Outcomes in Clinical Trials

Treatment outcomes in HS clinical trials have evolved greatly, leading to many scores that do not predictably correlate with each other. Although many trials use several objective scores when evaluating disease improvement, others may not. Understanding what each score measures can help clinicians to better interpret what gains can be made by implementing different treatment options in their patients with moderate-to-severe HS (see Table 1). Although this list is not exhaustive, these outcome measures were the most used in the articles reviewed from this scoping review.

**Table 1.** Summary of Treatment Outcomes in HS.

Outcome	Description	Clinician or patient reported
HiSCR	HiSCR is achieved if a patient has a ≥50% reduction in inflammatory lesion count (abscesses + inflammatory nodules), and no increase in abscesses or draining fistulas when compared with baseline. HiSCR can be adapted to be more stringent (eg, HiSCR75) or more lenient (HiSCR25), which corresponds to a 75% or 25% reduction, respectively.	Clinician
IHS4	IHS4 is a score that can be assessed at separate times. IHS4 = Nodules + 2(Abscesses) + 4 (Draining Fistulae). A ≥55% improvement in IHS4 score between two visits is considered a responder to treatment.	Clinician
HS-PGA	HS-PGA incorporates the number of abscesses, draining fistulas, inflammatory nodules, and non-inflammatory. Can be scored from 1 (clear) to 6 (very severe). Responders to treatment are defined as an HS-PGA of 1-2 with at least a 2-point improvement from baseline.	Clinician
HIDRAscore	HIDRAscore incorporates the number of inflammatory nodules, abscesses, fistulae, subumbilical lesions, and the HIDRAdisk, a quality-of-life index specific to HS.	Clinician and patient
DLQI	DLQI is a non-specific tool that focuses on the burden of skin disease on a patient’s quality of life.	Patient
HiSQOL	HiSQOL is a quality-of-life tool that was validated specifically to assess the symptom burden in patients with HS.	Patient

Abbreviations: DLQI, Dermatology Life Quality Index; HiSCR, Hidradenitis Suppurativa Clinical Response; HiSQOL, Hidradenitis Suppurativa Quality of Life Score; HS, Hidradenitis Suppurativa; HS-PGA, Hidradenitis Suppurativa Physician Global Assessment; IHS4, International Hidradenitis Suppurativa Severity Score System.

The Hidradenitis Suppurativa Clinical Response (HiSCR) is currently the most used treatment outcome to assess response in HS clinical trials.^9,10^ The purpose of this score is to assess the same patient at 2 different time points to see if they achieved a ≥50% reduction in inflammatory lesion count (abscesses + inflammatory nodules), and no increase in abscesses or draining fistulas when compared with baseline. This is meant to give a dichotomous assessment of responder versus non-responder, which is useful in clinical trials. The HiSCR has since been adapted to include other cut-offs to make a more lenient (HiSCR25; ≥25% reduction) or more stringent (HiSCR75; ≥75% reduction) outcome.

Another commonly used outcome is the International Hidradenitis Suppurativa Severity Score System (IHS4).^11^ This score shares the same items as the HiSCR, in a simple formula:

ISH4 = Nodules + 2(Abscesses) + 4(Draining fistulae)

This score can be used as a severity measure or a treatment outcome by considering patients with ≥55% improvement in IHS4 score between 2 visits as responders to treatment.

Similarly, the HS physician’s global assessment scale (HS-PGA) can be used as a severity measure and a treatment outcome. This score incorporates 4 items: the number of abscesses, draining fistulas, inflammatory nodules, and the presence/absence of non-inflammatory nodules. It has been used as a treatment outcome by defining responders as patients achieving an HS-PGA score of clear, minimal, or mild with at least a 2-grade improvement relative to baseline.

Although these scores are useful, they lack useful patient-reported input. The HIDRAscore is another outcome used in some clinical trials that incorporates the number of inflammatory nodules, abscesses, and draining fistulas, the presence or absence of subumbilical lesions, and the HIDRAdisk, a quality-of-life index specific to HS.^12,13^

The Dermatology Life Quality Index (DLQI) and Hidradenitis Suppurativa Quality of Life (HiSQOL) scores are both patient-reported tools that focus on the burden of disease on their quality of life.^14,15^ The DLQI is an older tool that is used for various dermatologic conditions. The HiSQOL assesses HS-specific health-related quality of life factors.

For this review, the main outcome that will be discussed is the HiSCR as it is the most used treatment outcome across clinical trials.

## Biologics in the Treatment of HS

### Tumour Necrosis Factor-α Inhibitors

#### Adalimumab

##### Efficacy and safety

Adalimumab is a recombinant monoclonal antibody that binds and neutralizes tumour necrosis factor (TNF)-α.^16^ It is the most well-studied medication in the treatment of moderate-to-severe HS, and for a long time, it was the only medication indicated for this use. The highest level of evidence of adalimumab’s efficacy and safety in moderate-to-severe HS comes from the PIONEER I and PIONEER II trials.^17^ These are phase 3, multicentre, placebo-controlled randomized clinical trials (RCT) that evaluated the efficacy and safety of adalimumab weekly for 12 weeks versus placebo.^17^ After the initial investigatory period, those who received adalimumab for 12 weeks were further randomized to continue to receive adalimumab weekly, adalimumab every other week or placebo for another 24 weeks.^17^ In these trials of 307 and 326 patients, clinical response rates at week 12 were significantly higher for the groups receiving adalimumab weekly than for the placebo groups: 41.8% versus 26.0% in PIONEER I and 58.9% versus 27.6% in PIONEER II.^17^ Among the patients who achieved the primary outcome at week 12, the advantage of continuing treatment with adalimumab weekly or every other week compared to placebo was not immediately evident in either trial.^17^ However, this initial interpretation was reconsidered after a pooled analysis of the results from the 2 PIONEER trials was conducted, aiming for a more comprehensive assessment of outcomes. This analysis suggested that weekly adalimumab treatment may indeed be the most effective maintenance dosing regimen in the medium term.^18^ In these trials, no new safety signals were found in the HS population (see Table 2 for a summary on biologics used in HS).^17^

**Table 2.** Summary of Biologics in HS.

Biologic	Mechanism of action	Efficacy	Safety	Monitoring	Highest level of evidence
TNF-α inhibitors					
Adalimumab 160 mg SC on day 0, 80 mg SC on day 15, then 40 mg SC weekly or 80 mg SC every 2 weeks thereafter	Recombinant monoclonal antibody that binds and neutralized human TNFα.	Primary endpoint of HiSCR at week 12: Adalimumab, 41.8% and 58.9%; PBO, 26% and 27.6%.	Injection site reaction, infection, headache.	Baseline: CBC, LFTs, creatinine, TB screen, viral hepatitis Ongoing: LFTs, skin examinations, signs and symptoms of infections, heart failure, and malignancy.	Phase 3 RCTs^17^
Infliximab 5-10 mg/kg IV infusion at weeks 0, 2, and 6, followed by every 8 weeks thereafter	Chimeric monoclonal antibody that binds to and neutralizes human TNFα.	Primary endpoint of >50% reduction in Hidradenitis Suppurativa Severity Index score at 8 weeks: Infliximab group: 27% PBO: 5%. Systematic review and meta-analysis: 87% response rate	Infection, infusion reaction, headache	Baseline: CBC, LFTs, creatinine, TB screen, viral hepatitis Ongoing: LFTs, skin examinations, signs and symptoms of infections, heart failure, and malignancy.	RCT and Systematic review and meta-analysis.^48,60^
Etanercept 50 mg SC once to twice weekly	TNF-α inhibitor that acts as a soluble TNF receptor and binds to free TNF-α and TNF-ß.	No significant difference in PGA compared to placebo at week 12 or 24.	Infection, injection site reaction	Baseline: CBC, LFTs, creatinine, TB screen, viral hepatitis. Ongoing: LFTs, skin examinations, signs and symptoms of infections, heart failure, and malignancy.	RCT^62^
Certolizumab 400 mg SC every 2 weeks	Pegylated humanized antibody Fab’ fragment of TNF-α monoclonal antibody.	Case reports suggest efficacy in moderate-to-severe HS.	Likely to be safe during pregnancy. Infection, antibody development, nausea.	Baseline: CBC, LFTs, creatinine, TB screen, viral hepatitis. Ongoing: LFTs, skin examinations, signs and symptoms of infections, heart failure, malignancy.	Case studies^71-76^
IL-17 Inhibitors					
Secukinumab 300 mg SC at weeks 0, 1, 2, 3, and 4, and every 4 weeks thereafter with the option to increase to every 2 weeks	Monoclonal antibody that selectively binds and inhibits IL-17a.	Primary endpoint of HiSCR at week 16: Every 2 weeks, 45% and 42%; Every 4 weeks, 42% and 46%; PBO, 34% and 31%.	Headache, upper respiratory tract infection, IBD	Baseline: CBC, LFTs, creatinine, TB screen, viral hepatitis, signs and symptoms of IBD. Ongoing: LFTs, skin examinations, signs and symptoms of infections or IBD.	Phase 3 RCTs^83^
Bimekizumab 640 mg SC at week 0, and 320 mg every 2 weeks thereafter	Monoclonal antibody that selectively binds and inhibits IL-17A, IL-17F, and IL17-AF.	Primary endpoint of HiSCR at week 16: Every 2 weeks, 47.8% and 52%; Every 4 weeks, 45.3% and 53.8%; PBO, 28.7% and 32.2%.	Headache, oral candidiasis, diarrhoea, infections	Baseline: CBC, LFTs, creatinine, TB screen, viral hepatitis, signs and symptoms of IBD, depression and suicidal ideation. Ongoing: LFTs, skin examinations, signs and symptoms of IBD, depression or suicidal ideation.	Phase 3 RCTs^86-88^
Sonelokimab 120 to 240 mg SC at weeks 0, 2, and 4, then every 4 weeks thereafter	Humanized Nanobody that selectively binds IL-17A and IL-17F, as well as human albumin to enrich sonelokimab at sites of inflammation.	Primary endpoint of HiSCR75 at week 12: SLK 120 mg, 43.3%; SLK 240 mg, N.D.; PBO, 14.7%.	Upper respiratory tract infection, injection site reaction, oral candidiasis	Baseline: CBC, LFTs, creatinine, TB screen, viral hepatitis, signs and symptoms of IBD.^a^ Ongoing: LFTs, skin examinations, signs and symptoms of infections or IBD.^a^	Phase 2 RCT^89,90^
Ixekizumab 160 mg SC at week 0, followed by 80 mg at weeks 2, 4, 6, 8, 10, and 12, then ever 4 weeks thereafter	Monoclonal antibody that selectively binds and inhibits IL-17A.	Case reports suggesting efficacy in moderate-to-severe HS.	Upper respiratory tract infection, injection site reaction, IBD, oral candidiasis	Baseline: CBC, LFTs, creatinine, TB screen, viral hepatitis, signs and symptoms of IBD. Ongoing: LFTs, skin examinations, signs and symptoms of infections or IBD.	Case studies/ series^92,93^
Brodalumab 210 mg SC every week or every 2 weeks	Monoclonal IgG2 antibody directed at the IL-17RA receptor.	Primary endpoint of HiSCR at week 12: 10 out of 10 patients on brodalumab.	Upper respiratory tract infection, arthralgia, tinea infections	Baseline: CBC, LFTs, creatinine, TB screen, viral hepatitis, signs and symptoms of IBD. Ongoing: LFTs, skin examinations, signs and symptoms of infections or IBD.	Open-label cohort studies^99,100^
IL-1 inhibitors					
Anakinra 100 mg SC once daily	IL-1 receptor antagonist.	Primary composite endpoint of decreased disease activity at week 12: Anakinra 7 of 9 (78%); PBO 2 of 10 (20%).	Infection, injection site reaction, hypernatremia, constipation	Baseline: CBC, creatinine, sign and symptoms of injections Ongoing: CBC	RCT^104^
Canakinumab 150 mg SC monthly	Monoclonal antibody that binds and interferes with IL-1.	Mixed results of efficacy based on case studies	Infection, diarrhoea, increased uric acid, headache	Baseline: CBC, CRP, serum amyloid A protein A, sign and symptoms of infection	Case studies^110-112^
Bermekimab 7.5 mg/kg IV infusion every 14 days Or 400 mg SC weekly	Monoclonal antibody that binds and interferes with IL-1α.	Primary endpoint of HiSCR at week 12: Bermekimab, 60%; PBO, 10%.	Injection site reaction, HS exacerbation	No recommendation as of yet.	RCT^114^
Lutikizumab 300 mg SC weekly or every other week	Monoclonal antibody that acts as a dual-variable-domain IL-1α/1β antagonist.	Primary endpoint of HiSCR at week 16: Weekly, 48.7%; Every other week, 59.5%, PBO, 35%	Diarrhoea, headache, naso-pharyngitis	No recommendation as of yet.	Phase 2 RCT^116^
IL-12/23 inhibitors					
Ustekinumab Weight-adjusted induction as a single IV dose or weight-adjusted SC induction at 0 and 4 weeks, then 45 or 90 mg SC every 12 weeks thereafter	Monoclonal antibody that binds to and interferes with IL-12 and IL-23.	Primary endpoint of HiSCR at week 16 and 40, respectively: 7 of 14 (50%), and 8 of 17 (47%) on ustekinumab	Infection, nausea, injection site reaction	Baseline: CBC, LFTs, creatinine, TB screen, viral hepatitis, signs and symptoms of posterior reversible encephalopathy syndrome. Ongoing: LFTs, skin examinations, signs and symptoms of infections.	Open-label cohort studies^123,124^

Abbreviations: CBC, complete blood count; HiSCR, Hidradenitis Suppurativa Clinical Response; HS, Hidradenitis Suppurativa; IBD, inflammatory bowel disease; IgG, immunoglobulin G; IL, interleukin; LFT, liver function test; PBO, placebo; SC, subcutaneously; TB, tuberculosis; TNF, tumour necrosis factor.

a Monitoring parameters extrapolated from recommendations of other IL-17 inhibitors as there are no recommendations yet for monitoring sonelokimab as it is not yet marketed.

The results of these PIONEER studies have been corroborated and similar efficacy and safety results have been found in other real-world safety, effectiveness, and long-term efficacy studies.^19-25^ Additionally, in the 3-year open-label extension study of the PIONEER trials, a sustained response is seen through week 168 in 52.3% of patients treated with adalimumab 40 mg weekly.^25^ These studies expanded upon the PIONEER trials by showing that adalimumab is an effective long-term option for moderate-to-severe HS in clinical trials and real-world settings.

Although adalimumab has been shown to be effective at 40 mg weekly, it was hypothesized that dose intensification could be an effective strategy to increase response rates in certain populations.^26-28^ Although there are mixed results from these studies, dose intensification to up to 80 mg per week may be beneficial in patients who have lost response to adalimumab over time at standard doses.

##### Predictors of efficacy and drug survival

Although the PIONEER studies have shown notable efficacy in moderate-to-severe HS, about 1 in 2 patients were non-responders in these trials. Many studies have investigated what factors can predict successful treatment of adalimumab and prolong drug survival in this population.^29-33^ One of the demonstrable factors that can impact the efficacy of adalimumab is the continuation of tetracycline antibiotics.^17^ In the PIONEER II trial, 19% of patients continued their baseline oral antibiotic, whereas in the PIONEER I trial, patients were required to stop treatment with oral antibiotics for at least 28 days before entering the trial. The calculated number needed to treat from PIONEER 1 is 6.3, whereas the number needed to treat from PIONEER 2 is 3.2.^17^ Although the authors concluded that responses to adalimumab were similar regardless of whether baseline antibiotic therapy was continued, this difference in the number needed to treat cannot be ignored. It has been found that patients with a body mass index (BMI) greater than 30 have demonstrated signs of both clinical and physiological deterioration with elevated HS-PGA, pain scores, and inflammatory markers while on adalimumab compared to those with a BMI less than 30.^29^ As discussed earlier, dose intensification may be a strategy to overcome this.^26^ Furthermore, therapeutic delay was correlated to a lack of response to adalimumab in a real-life retrospective multicentre study of 389 patients treated with adalimumab.^31^ This inverse correlation between the therapeutic delay and clinical response supports early adalimumab use in HS treatment.^31^

Another key discussion topic for patients is the factors influencing drug survival and flare prevention in HS patients on adalimumab. In a study of 116 patients with HS on adalimumab, the addition of oral immunosuppressants like methotrexate and mycophenolate did not significantly prolong the duration of therapy with adalimumab, and these patients were just as likely to discontinue adalimumab as those on monotherapy.^30^ Another retrospective study of 115 patients found that the only modifiable risk factor associated with disease progression and recurrence of flares was therapeutic delay.^32^

##### Drug monitoring

Although there is strong evidence to support the utility of drug monitoring of adalimumab in patients with inflammatory bowel disease (IBD), there is minimal guidance in patients with HS. An algorithm for drug monitoring of adalimumab in patients with HS has been proposed; however, there are no studies validating its efficacy or utility.^34^ Others have suggested that higher levels of TNF-α inhibitors may be required in the treatment of HS, similar to patients with IBD.^35-37^ Regular drug monitoring of adalimumab in HS is still in its infancy, and it is expected that our understanding of optimal levels and how to interpret these results will be elucidated in the near future.

##### Biosimilars

Recently, biosimilar medications have been made available and are starting to replace originator biological products in many countries. As they are generally more affordable, formularies preferentially cover biosimilars over branded products. Providers and patients often question the efficacy and safety of biosimilars as they are not required to undergo clinical trial testing before entering the market. Several studies have investigated the effect of switching from originator adalimumab to biosimilar products.^38-42^ All of these studies were retrospective analyses of HS patients following patients who were switched from originator adalimumab to various biosimilars. The efficacy and safety from these results are heterogeneous; however, there is a trend toward higher discontinuation rates and higher drug failure of biosimilars compared to the originator.^39-41^ These results need to be interpreted with caution as the patients were aware of the switch from branded to biosimilar products, which can significantly affect their perception of efficacy and safety. Further larger-scale, blinded studies are required to elucidate the real-world effectiveness and safety of biosimilar products compared to the originator.

#### Infliximab

Infliximab is a chimeric immunoglobulin G (IgG) monoclonal antibody that binds and neutralizes TNF-α.^43^ It is only administered intravenously, compared to most other TNF-α inhibitors which can be administered subcutaneously (SC). Its use in the treatment of moderate-to-severe HS has been widely investigated in an RCT and several prospective and retrospective observational studies.^44-59^ A systematic review and meta-analysis evaluated 19 articles investigating the use of infliximab in moderate-to-severe HS that met the pre-determined inclusion criteria (6 prospective and 13 retrospective).^60^ The included articles used many outcomes to determine efficacy including physician assessment, HiSCR, HS-PGA, Hidradenitis suppurativa score, and Hidradenitis Suppurativa Severity Index (HSSI). Based on this meta-analysis, the pooled response rate was 83%.^60^ These results should be interpreted with caution as depending on the outcomes used in the various studies, the threshold to achieve “responder” can be quite different. Compared to the results of the 1 RCT that was included in this review, the meta-analysis seems to heavily favour the infliximab arm. Notably, this systematic review did not use the same primary outcome that was reported in the RCT. In this phase 2 RCT, patients with moderate-to-severe HS were randomized to receive infliximab or placebo for 8 weeks.^48^ A numerically greater number of participants achieved the primary endpoint of a >50% reduction in HSSI in the infliximab group (27%) compared to the placebo group (5%).^48^ However, the systematic review reported that the response rate from this RCT was 87%, corresponding to the percentage in the infliximab group that achieved >25% reduction from the baseline HSSI score. This highlights one of the limitations of relying on systematic reviews when the outcome measures from the included studies differ significantly. The most common adverse events in these studies were non-serious infections, secondary infections of HS, infusion reactions, and headaches. The most common dose used in trials was 5 mg/kg intravenous (IV) infusion; however, a retrospective cohort study suggests that doses of up to 7.5 to 10 mg/kg IV infusion every 4 to 6 weeks were well tolerated and led to clinical improvements in patients with HS.^45^

In light of these results, infliximab has been included in both the European and North American guidelines as an effective treatment option for moderate-to-severe HS and is often used after treatment failure with adalimumab.^6,7^

#### Etanercept

Etanercept is another TNF-α inhibitor that acts as a soluble TNF receptor and binds to free TNF-α and TNF-ß.^61^ Its efficacy in moderate-to-severe HS has not been as well studied compared to adalimumab and infliximab and the available results are not as promising. In a single-centre, placebo-controlled RCT of 20 patients with moderate-to-severe HS, there was no statistical difference in physician global assessment, patient global assessment and DLQI at week 12 or 24 between the etanercept and placebo group.^62^ Furthermore, there have been several open-label trials and case series evaluating the efficacy of etanercept with response rates ranging from 20% to 60%.^63-66^ It should be noted that these results were published before the development and validation of the HiSCR, so these studies relied on outcomes such as DLQI, PGA, and Sartorius scores. Notably, there is also a case report of a patient with HS and psoriasis who was treated with etanercept and experienced an HS flare with an increase in suppurative lesions.^67^ The available evidence for etanercept in moderate-to-severe HS does not demonstrate that it should be used routinely, as there are other options in the TNF-α inhibitor class with higher level evidence of benefit.

#### Certolizumab

Certolizumab pegol is a recombinant humanized PEGylated antibody Fab’ fragment, which specifically binds and neutralizes TNF-α.^68^ The main differentiator between certolizumab and other TNF-α inhibitors is its absence of the fragment crystallizable region, which prevents active placental transfer.^69^ In a cohort pharmacovigilance study, reports from 1137 prospectively reported pregnancies with maternal exposure to certolizumab showed no teratogenic effect or increased risk of foetal death compared to the general population.^70^ Due to its unique pharmacokinetic properties, certolizumab is an option used in women requiring biologic therapy while pregnant. For this reason, there are a disproportionate number of case studies showing its efficacy and safety in women who are pregnant compared to the general population. Two groups report the cases of 3 patients who underwent successful treatment with certolizumab while pregnant, with the improvement of symptoms at 2 months for 2 of the women, and 3 months for the other.^71,72^ All 3 cases achieved HiSCR. Certolizumab has also been reported to be an effective option in the general population of patients with moderate-to-severe HS with case studies reporting its efficacy in those with difficult-to-control HS, with concomitant inflammatory immune-mediated diseases such as Crohn’s disease (CD) and psoriasis, and with treatment failure with adalimumab.^73-76^ Further large-scale studies are necessary to elucidate the safety and efficacy of certolizumab in patients with moderate-to-severe HS, particularly in those of childbearing ability as a potential option during pregnancy.

### Interleukin-17 Inhibitors

#### Secukinumab

Secukinumab is a human IgG1 monoclonal antibody that selectively binds interleukin (IL)-17A cytokine to inhibit its interaction with the IL-17 receptor.^77^ Shortly after secukinumab was brought to market for its use in plaque psoriasis in 2015, several case reports, retrospective observational studies, and open-label exploratory trials have shown its promising efficacy for the treatment of moderate-to-severe HS with HiSCR results ranging from 48.9% to 67%.^78-82^

More recently, 2 phase 3, multicentre, placebo-controlled RCTs entitled the SUNSHINE and SUNRISE trials investigated the use of secukinumab in moderate-to-severe HS.^83^ In the SUNSHINE and SUNRISE trials, 541 and 543 participants were included in the analysis. The primary endpoint was the proportion of patients achieving HiSCR at week 16. Both trials had 3 treatment arms, consisting of the secukinumab every 2 weeks, secukinumab every 4 weeks, and placebo. In both trials, significantly more participants in the secukinumab every 2 weeks group achieved the primary endpoint compared to placebo. In the SUNRISE trial, but not the SUNSHINE trial, significantly more participants in the secukinumab every 4 weeks group achieved the primary endpoint compared to placebo. In both trials, the onset of efficacy was as rapid as within the first 4 weeks. Secukinumab every 2 weeks also showed improvement in percentage change from baseline abscess and inflammatory nodule (AN) count, reduction in flares, higher Numerical Rating Scale 30 (NRS30) response, and improved DLQI. Furthermore, in both trials, the clinical efficacy observed at week 16 was sustained and improved over time to week 52. In both trials, secukinumab was well tolerated with no new safety signals identified in this population.

Following these trials’ promising results, secukinumab was approved by the Food and Drug Administration and Health Canada for use in moderate-to-severe HS in the USA and Canada, marking the second biologic to be approved for this indication.^77^ This will increase access to biologic therapy for patients with HS and make secukinumab a viable first-line option.

#### Bimekizumab

Like secukinumab, bimekizumab is a humanized IgG1/k monoclonal antibody, however, it binds and neutralizes IL-17A, IL-17F, and IL-17AF cytokines.^84^ Currently, bimekizumab is marketed for use in moderate-to-severe plaque psoriasis; however, it has recently undergone phase 2 and phase 3 trials to show its efficacy and safety in HS. In a phase 2, multicentre, placebo-controlled RCT of 90 patients, patients were randomized to receive bimekizumab every 2 weeks, placebo, or adalimumab in a 2:1:1 design.^85^ The primary endpoint was the proportion of patients who achieved HiSCR at week 12. At week 12, significantly more patients in the bimekizumab group achieved the primary endpoint as well as higher HiSCR75, higher HiSCR90, improved IHS4, lesser skin pain, and improved DLQI scores. Although this trial was not designed to assess bimekizumab versus adalimumab, a numerically greater number of patients on bimekizumab achieved HiSCR75 (46% vs 35%), and HiSCR90 (32% vs 15%) compared to adalimumab. Both the adalimumab and bimekizumab groups showed improvements as early as week 2 compared to placebo.

Furthermore, bimekizumab was also investigated in the BE-HEARD I and BE HEARD II phase 3, multicentre, placebo-controlled, RCTs of 505 and 509 patients, respectively.^86^ Similar to the trials of secukinumab, both of these trials evaluated 2 dosing regiments of bimekizumab (every 2 weeks and every 4 weeks) versus placebo. A significantly higher proportion of patients in the bimekizumab every 2 weeks group achieved the primary endpoint of HiSCR at week 16 in both trials. Although a numerically greater number of patients in the bimekizumab every 4 weeks group achieved HiSCR, only the BE HEARD II trial achieved statistical significance. In keeping with previous results, significantly more patients receiving bimekizumab every 2 weeks also achieved HiSCR75 compared to placebo. These clinical responses were maintained at week 48. In addition, results from a presentation at Symposium on Hidradenitis Suppurativa Advances 2023 showed that treatment with bimekizumab resulted in greater improvements in HiSQOL total score and skin pain compared to placebo.^87,88^ The safety profile of bimekizumab in these trials was consistent with previous studies, with no new safety signals observed.

#### Sonelokimab

Sonelokimab is an investigational Nanobody that selectively binds and neutralizes IL-17A and IL-17F, as well as binds to human albumin, which facilitates enrichment of sonelokimab at sites of inflammation.^89,90^ Sonelokimab is not currently marketed for a specific indication; however, it has undergone clinical trials in both plaque psoriasis and HS. Results from a phase 2, multicentre, placebo-controlled, RCT, entitled the MIRA trial, were presented at European Academy of Dermatology and Venereology 2023.^89,90^ 234 patients were recruited to evaluate 2 different doses of sonelokimab versus placebo and adalimumab as an active reference arm. A significantly higher proportion of patients on sonelokimab 120 mg every 4 weeks (43.3%) compared to placebo (14.7%) achieved HiSCR75 at week 12, which is a more stringent primary endpoint compared to other clinical trials.^89^ Furthermore, maintenance treatment with sonelokimab showed that 57% of patients achieved HiSCR75 at week 24.^90^ Interestingly, the 120 mg dose appeared to be numerically superior to the 240-mg dose across the 24 weeks of the trial. For non-responders to adalimumab at week 12 switching to sonelokimab resulted in HiSCR75 response rates like those randomized to sonelokimab at baseline. Sonelokimab also led to significant improvements in HiSCR, HiSCR90, IHS4, patient-reported quality of life, skin pain, and HS symptoms compared with placebo. Safety results of sonelokimab were consistent with previously reported studies with no new observed signals, although these results have only released via presentation and press releases. The MIRA trial adds to the existing knowledge that IL-17 is involved in the pathophysiology of HS and stands out as the only trial to use HiSCR75 as the primary outcome.

#### Ixekizumab

Ixekizumab is a humanized IgG monoclonal antibody that selectively binds to IL-17A, which prevents it from binding to the IL-17 receptor.^91^ No clinical studies to date have been conducted to evaluate its use in patients with moderate-to-severe HS. Only a few case studies and case series have been published.^92,93^ In 1 report, a 50-year-old man with severe psoriasis and Hurley stage II HS underwent treatment with ixekizumab, which led to significant improvement of both conditions at week 10.^93^ Furthermore, in a case series of HS patients who were resistant to conventional treatments and adalimumab were treated with ixekizumab, 4 out of the 5 patients achieved HiSCR at 12 weeks, suggesting that ixekizumab may be effective for HS in challenging cases.^92^

#### Brodalumab

Brodalumab is a human monoclonal IgG2 antibody directed at the IL-17RA receptor, unlike other IL-17 inhibitors that target the IL-17A ligand.^94^ Like ixekizumab, there are no RCTs evaluating its use in moderate-to-severe HS. Numerous case studies and case series are showing successful treatment of HS with brodalumab after failure of TNF inhibitors or with concomitant psoriasis.^95-98^ Two open-label cohort studies designed to assess the efficacy and safety of brodalumab in patients with moderate-to-severe HS at 2 different administration schedules both demonstrated that 10 out of 10 patients achieved HiSCR at week 12.^99,100^ Furthermore, weekly administration of brodalumab showed that 80% achieved HiSCR75, and 50% achieved HiSCR100.^100^ In addition, data from these studies showed that expression of LCN2 in skin or IL-17A in serum may be used as biomarkers to stratify patients that may have a superior molecular response to brodalumab.^101^ Given these promising preliminary results, larger RCTs are needed to further elucidate brodalumab’s efficacy in moderate-to-severe HS.

### IL-1 Inhibitors

#### Anakinra

The IL-1 pathway has been demonstrated to be involved in the pathogenesis of HS and represents another possible target for treatment.^102^ Anakinra is a small molecule IL-1 receptor antagonist.^103^ Its safety and efficacy have been evaluated in several case studies with mixed results, an open-label study, and 1 placebo-controlled RCT.^104-108^ This trial of 20 patients with Hurley stage II or III disease were randomized to receive 100 mg of anakinra or placebo for 12 weeks, with an observation period of 24 weeks.^104^ Seven of 9 (78%) patients in the anakinra group achieved the primary composite endpoint of decreased disease activity as measured by the differences of Visual Analogue Scale (VAS), severity scores and DLQI at week 12, compared to 2 of 10 (20%) patients in the placebo group. The HiSCR at week 12 was retrospectively assessed and found similar results to the primary endpoint. The efficacy of anakinra was sustained until week 16, although the disease activity score gradually rose after week 16. In an open-label study, discontinuation of anakinra led to rebound in 4 of the 5 patients who completed the study.^104,105^ Anakinra appears to be an effective off-label option for moderate-to-severe HS; however, some limitations to use in everyday practice include the small sample size in these trials and daily SC injections.

#### Canakinumab

Canakinumab is a human monoclonal antibody that binds the IL-1 cytokine and prevents interaction with cell surface receptors.^109^ There are mixed results about the efficacy of canakinumab in patients with moderate-to-severe HS based on several case reports.^110-112^

#### Bermekimab (MABp1)

Bermekimab is another human monoclonal antibody that neutralizes IL-1α by binding and neutralizing the cytokine.^113^ The efficacy of bermekimab was evaluated in a randomized, placebo-controlled clinical trial of 20 patients with Hurley stage II or III disease.^114^ Patients were randomized to receive 7.5 mg/kg of bermekimab as an IV infusion or matched placebo. A total of 60% of patients receiving bermekimab achieved the primary endpoint of HiSCR at week 12 compared with 10% in the placebo group. The odds ratio of positive HiSCR under bermekimab was 13.5. Those in the placebo group were allowed to participate in an open-label extension and receive bermekimab.^115^ Of the 8 participants who participated in the open-label extension, 6 achieved HiSCR at week 12 of treatment, which is consistent with the original trial.

In addition, a phase 2, multicentre, open-label study split 42 patients with HS into 2 groups based on whether they previously failed anti-TNF therapy or not. Both groups received 400 mg of bermekimab SC each week for 12 weeks.^113^ The primary endpoint was the safety and tolerability of bermekimab. Both groups achieved similar efficacy results of HiSCR 63% and 61% for the TNF failure group and TNF naïve group, respectively. Further blinded studies are required to further evaluate the efficacy of bermekimab and to elucidate the optimal route of administration.

#### Lutikizumab

Lutikizumab is an investigational, dual-variable-domain IL-1α/1β antagonist.^116,117^ The efficacy and safety of lutikizumab were assessed in a phase 2, placebo-controlled RCT of 153 patients with Hurley stage 3 HS who had previously failed TNF-α inhibitors.^116^ Patients were randomized to receive lutikizumab 100 mg every other week, 300 mg every other week, 300 mg every week, or placebo. More patients in all lutikizumab groups achieved the primary endpoint of HiSCR and more patients in the lutikizumab 300 mg every other week and weekly groups achieved secondary endpoints of NRS30 and HiSCR75 compared to placebo. Further phase 3 studies are underway to evaluate the efficacy and safety of lutikizumab with a larger sample size.

### IL-12/IL-23 and IL-23 Inhibitors

#### Ustekinumab

Ustekinumab is a human monoclonal antibody with a unique mechanism. It inhibits the p40 subunit of the IL-12 and IL-23 cytokines, thereby preventing their interaction with the IL-12Rß1 receptor.^118^ Although there are no RCTs investigating ustekinumab for use in patients with moderate-to-severe HS, there are numerous published case series and retrospective studies showing positive results.^119-122^ In 1 open-label study evaluating 45 mg or 90 mg of ustekinumab in patients with moderate-to-severe HS, 14 of 17 patients achieved a moderate-to-marked improvement of the modified Sartorius score and 8 of 17 patients achieved HiSCR at week 40.^123^ Furthermore, another single-arm study investigated high-dose IV ustekinumab induction followed by SC dosing in HS patients who had failed other biologic therapy.^124^ In this study, 7 of the 14 patients enrolled achieved HiSCR at week 16.^124^ Despite the difficulty in establishing the efficacy and safety of ustekinumab given the sampling number and lack of properly controlled studies, it remains a second-line off-label option for moderate-to-severe HS.

#### Other IL-23 inhibitors

In addition to ustekinumab, other IL-23 inhibitors have been investigated for use in moderate-to-severe HS. Despite promising case studies showing variable efficacy with risankizumab, a phase 2 RCT of 243 patients investigating risankizumab at 2 doses versus placebo failed to meet the primary endpoint of HiSCR at week 16, and the trial was terminated early.^125-128^

Similarly, in a phase 2 RCT of 184 patients investigating guselkumab SC, guselkumab IV induction then switched to guselkumab SC, and placebo failed to show a statistically significant difference in the primary endpoint of HiSCR at week 16, despite numerically higher HiSCR scores.^129^

## Small Molecule Immunomodulators in the Treatment of HS

### Janus Kinase Inhibitors

#### Povorcitinib (INCB054707)

Povorcitinib, previously INCB054707, is a small-molecule Janus kinase 1 (JAK1) inhibitor with approximately 52-fold greater selectivity for JAK1 versus JAK2.^130^ This mechanism lends well to reducing cytokine signalling involved in HS pathogenesis while limiting JAK2-mediated side effects.^130,131^ In a phase 2, multicentre, placebo-controlled, RCT of 209 patients investigating 3 doses of povorcitinib, all 3 povorcitinib-treated groups achieved the primary endpoint of significantly reduced mean change from baseline in AN count compared to placebo at week 16.^132^ In addition, more povorcitinib-treated patients achieved the secondary endpoint of HiSCR at week 16 compared to placebo. The 45 and 75 mg doses showed statistical differences in reduced mean change in AN and HiSCR as early as week 6 and week 2, respectively, compared to placebo. This trial also showed that a numerically larger percentage of patients receiving any dose of povorcitinib also achieved HiSCR75/HiSCR90/HiSCR100 and IHS4-55/IHS4-75/IHS4-90/IHS4-100. In addition, there was a numerically greater improvement in HiSQOL and DLQI, especially in the 45 and 75 mg groups. In a press release announcing the results of the open-label extension of this trial, all groups were switched to povorcitinib 75 mg and had sustained efficacy up to week 52.^133^

Furthermore, 2 smaller multicentre phase 2 trials, of 10 and 35 participants, primarily investigating the safety of povorcitinib in patients with moderate-to-severe HS showed similar efficacy results with rapid and dose-dependent clinical responses.^130^ While numerically, these results show positive responses with the use of povorcitinib, these results should be interpreted with caution as this study was not powered to assess for efficacy and there is a significantly high response rate in the placebo group, which underscores the need for trials with longer follow up and larger sample sizes.^130,134^ In all 3 studies, povorcitinib was well-tolerated with few treatment-emergent adverse effects and no concerning safety signals. Overall, despite not currently being approved for the treatment of moderate-to-severe HS, povorcitinib is generally efficacious and well tolerated across doses (see Table 3 for a summary of small molecule immunomodulators used in HS).

**Table 3.** Summary of Small Molecule Immunomodulators in HS.

Immunomodulator	Mechanism of action	Efficacy	Safety	Monitoring	Highest level of evidence
JAK inhibitors					
Povorcitinib 15, 45, or 75 mg PO once daily	Selective JAK1 inhibitor	Primary endpoint of reduced AN count from baseline achieved at all doses of povorcitinib at week 16. Significantly higher proportion of povorcitinib-treated patients achieved HiSCR compared to placebo at week 16: 15 mg, 48.1%; 45 mg, 44.2%; 75 mg, 45.3%; PBO, 28.2%.	Fatigue, headache, upper respiratory tract infection, thrombocytopenia.	Baseline: CBC, LFTs, lipid panel, viral hepatitis, TB screen.^a^ Ongoing: CBC, LFTs, lipid panel, signs and symptoms of infection, skin examinations, symptoms of thrombosis.^a^	Phase 2 RCTs^130,132^
Upadacitinib 30 mg PO once daily	Selective JAK1 inhibitor	Primary endpoint of HiSCR compared to placebo at week 12: 30 mg, 38.3%; PBO, 23.8%.	Acne vulgaris, infections.	Baseline: CBC, LFTs, lipid panel, viral hepatitis, TB screen. Ongoing: CBC, LFTs, lipid panel, signs and symptoms of infection, skin examinations, symptoms of thrombosis.	Phase 2 RCT^137^
Tofacitinib 5 mg PO twice daily	Selective JAK1 and JAK3 inhibitor	Use of tofacitinib in refractory HS led to remission of severe HS.	Elevated lipids, infections, bone marrow suppression.	Baseline: CBC, LFTs, lipid panel, creatinine, viral hepatitis, TB screen Ongoing: CBC, LFTs, lipid panel, creatinine, signs and symptoms of infection, skin examinations.	Case reports^139^
PDE4 inhibitors					
Apremilast 30 mg PO twice daily	Selective PDE4 inhibitor	Primary endpoint of HiSCR30 compared to placebo at week 16: 8 of 15 (53%) in the apremilast group versus 0 of 5 (0%) in the placebo group.	Diarrhoea, nausea, vomiting, anxiety, depression, weight loss with long-term use, upper respiratory tract infections.	Weight, creatinine, signs and symptoms of mood changes, depression, or suicidal ideation.	RCT^157^
Roflumilast 500 µg PO once daily	Selective PDE4 inhibitor	Use of roflumilast in refractory HS led to remission of severe HS.	Diarrhoea, nausea, vomiting, anxiety, depression, weight loss with long-term use, upper respiratory tract infections.	Weight, creatinine, signs and symptoms of mood changes, depression, or suicidal ideation.	Case report^148^
Complement receptor inhibitors					
Avacopan 30 mg PO twice daily	Oral C5aR antagonist	Avacopan did not achieve statistical significance in the overall participants of the trial. However, in the sub-group of Hurley stage III, HiSCR at week 12 was statistically significant: 30 mg BID, 42.6%; PBO, 22.2%	Hypertension, GI disturbance, hepatotoxicity, headache.	Baseline: LFTs, viral hepatitis. Ongoing: LFTs, signs and symptoms of infection.	Phase 2 RCT^152^
Vilobemilab 800 mg as an IV infusion twice per week on week 1, then weekly thereafter	IV C5aR antagonist	In a single-arm open-label trial, 9 of 12 (75%) of patients achieved HiSCR at day 50.	Respiratory tract infection, soft tissue infection, and increase of aspartate aminotransferase.	Signs and symptoms of new infection during treatment.	Phase 2a open-label trial^153^

Abbreviations: AN, abscess and inflammatory nodule; CBC, complete blood count; C5aR, complement 5a receptor; HiSCR, Hidradenitis Suppurativa Clinical Response; IV, intravenous; JAK, Janus kinase; LFT, liver function test; PBO, placebo; PDE4, phosphodiesterase; RCT, randomized controlled trial; TB, tuberculosis; HS, Hidradenitis Suppurativa.

a Monitoring parameters extrapolated from recommendations of other JAK inhibitors as there are no recommendations yet for monitoring povorcitinib as it is not yet marketed.

#### Upadacitinib

Upadacitinib is another JAK inhibitor with increased selectivity for JAK1 relative to JAK2, JAK3 and TYK2 subtypes.^135^ In a retrospective cohort study, 15 of 20 patients achieved HiSCR at week 4, which increased to 20 of 20 patients at week 12.^136^ There were also significant improvements in HiSCR75, IHS4, and DLQI.^136^ This study showed the potential of upadacitinib in the treatment of HS and prompted the need for larger, placebo-controlled trials to validate these observations.

These results were corroborated by a phase 2, multi-centre, placebo-controlled, RCT of 68 participants, which showed a significantly higher proportion of upadacitinib-treated patients achieved the primary endpoint of HiSCR when compared to placebo.^137^ Furthermore, the HiSCR response was maintained through week 40. This study revealed no new safety signals in the HS population and was generally well-tolerated in the trial with no major adverse cardiovascular events or venous thromboembolism. These studies show that upadacitinib may be a suitable alternative treatment option and bolster the role of JAK inhibitors in the treatment of HS.

#### Tofacitinib

Tofacitinib exerts its mechanism by selectively inhibiting the JAK1 and JAK3 enzymes.^138^ Its role in the treatment of HS is not well elucidated, and current evidence is limited to a case series of 2 patients treated with tofacitinib in addition to other therapies.^139^

### PDE4 Inhibitors

#### Apremilast

Apremilast is an oral small-molecule phosphodiesterase 4 (PDE4) inhibitor, currently approved for use in plaque psoriasis, psoriatic arthritis (PsA), and oral ulcers associated with Bechet disease. The inhibition of PDE4 causes a reduction in the production of many inflammatory cytokines, which decreases the inflammatory response. There are several case reports of successful treatment of HS in patients with and without comorbid plaque psoriasis and PsA.^140-143^ To date, there is 1 RCT and 1 open-label study evaluating the efficacy and safety of apremilast in patients with mild-to-moderate HS.^144,145^ In the open-label study evaluating apremilast in patients with mild-to-moderate HS, 13 of the 20 patients enrolled achieved the primary endpoint of HiSCR30 at week 16 and 24.^144^ There were also significant improvements in HiSCR, Sartorius score, PGA score, VAS pain score, and DLQI. In the RCT, 20 patients with moderate HS, defined as HS-PGA score of 3, were randomized in a 3:1 ratio to receive apremilast 30 mg twice daily, or placebo for 16 weeks.^145^ HiSCR was achieved by 8 of the 15 (53%) patients receiving apremilast compared to 0 of the 5 (0%) patients receiving placebo at week 16. Furthermore, the apremilast group showed significantly lower abscess and nodule count, NRS for pain, and itch over 16 weeks compared to placebo-treated patients. The responders in the apremilast group were followed in a 2-year continuation study. Half of these responders discontinued within the first year, and the other half continued and had achieved HiSCR at both the 1-year and 2-year follow-up visits compared to baseline.^146^ Reasons for discontinuation included pregnancy, nausea, and resolution of HS symptoms. In both trials, the most common adverse effects included gastrointestinal adverse effects and headaches. These studies suggest that apremilast may be an effective and safe option in the treatment of moderate HS and further larger studies are warranted.

#### Roflumilast

Roflumilast is another PDE4 inhibitor that is currently approved for use in patients with chronic obstructive pulmonary disease systemically and plaque psoriasis topically.^147^ There is 1 case report exploring the use of oral roflumilast in the treatment of a patient with severe HS and concomitant plaque psoriasis and obesity.^148^ After the failure of adalimumab and infliximab, monotherapy with oral roflumilast 500 µg once daily was initiated. At 3 months, his IHS4 score had decreased from 16 to 9, he had achieved complete clearance of his psoriasis and a 9-kg weight loss. Improvements in his IHS4 score were maintained after 5 months of treatment. Roflumilast represents another treatment option that warrants further evaluation for the treatment of moderate-to-severe HS.

### Complement Receptor Inhibitors

#### Avacopan

Avacopan is an oral complement 5a receptor (C5aR) antagonist that is currently marketed for the treatment of anti-neutrophil cytoplasmic autoantibody-associated vasculitis.^149^ The inhibition of C5aR Is thought to reduce inflammation mediated by the complement pathway and neutrophil recruitment. As circulating concentrations of complement factor C5A are significantly increased in HS, it has been hypothesized that this pathway could be a promising target in treated HS.^150^ A phase II, RCT of 398 participants evaluated avacopan in patients with moderate-to-severe HS with an inadequate response to antibiotics.^151,152^ Patients were randomized to receive avacopan 10 mg, avacopan 30 mg, or placebo in a 1:1:1 ratio for 12 weeks, followed by a blinded treatment period of avacopan 10 mg or avacopan 30 mg for an additional 24 weeks. The primary endpoint of HiSCR was not met in either arm of this trial, however, more patients in the subgroup of Hurley stage III achieved HiSCR with avacopan 30 mg compared with placebo.^151,152^ This trial did not reveal any new safety signals in the HS population, and the majority of adverse effects were mild to moderate. Additional studies are warranted to investigate the utility of avacopan specifically in the severe HS population; however, it may be another off-label treatment option for those with severe symptoms.

#### Vilobelimab (IFX-1)

Vilobelimab, also known as IFX-1, is a monoclonal IgG antibody that selectively binds and inhibits C5a.^153^ In a prospective, open-label, single-arm phase IIa study, vilobelimab was evaluated in 12 patients with Hurley stage III disease.^153^ In this trial, 9 of the 12 patients achieved HiSCR on day 50, and 10 of the 12 achieved HiSCR on day 134. Vilobelimab was well tolerated during the trial; the adverse events during the treatment period included HS exacerbations, respiratory tract infections, and an increase in aspartate aminotransferase. An ongoing SHINE phase II trial will further elucidate the efficacy of IFX-1.

## Combination Therapy for Refractory HS

Generally, the treatment of refractory HS involves optimizing current treatment or switching treatment strategies. Currently, there is minimal guidance about combining the medications reviewed above. While most of the trials described show that roughly 50% respond, this still leaves many without successful responses. Based on the current understanding of the immunopathogenesis of HS, some have suggested certain combination regimens that could theoretically be safe and effective including JAK inhibitor + IL-17 inhibitor, or TNF-α inhibitor + JAK inhibitor.^154^ While there are no formal studies investigating these combinations, 2 case studies exist showing successful treatment of patients with HS and comorbid CD with the combinations of guselkumab + apremilast and adalimumab and ustekinumab.^155,156^ Combination therapy may represent a novel treatment strategy that has not yet been investigated for moderate-to-severe HS.

## Conclusion

This review outlines the available evidence for the pharmacologic treatment of moderate-to-severe HS. As an overview, biologic medications including TNF-α inhibitors, IL-17 inhibitors, IL-1 inhibitors, and IL-12/23 inhibitors as well as oral medications including JAK inhibitors and PDE4 inhibitors have been investigated for use in patients with moderate-to-severe HS. Currently, only adalimumab and secukinumab are approved for this indication. The choice of agent to use largely depends on patient comorbidities and patient preferences. Although the body of evidence for these medications in moderate-to-severe HS is growing, further advocacy work is necessary to increase the number of on-label options to increase access to these treatments.
